# Human Serum Promotes Osteogenic Differentiation of Human Dental Pulp Stem Cells *In Vitro* and *In Vivo*

**DOI:** 10.1371/journal.pone.0050542

**Published:** 2012-11-29

**Authors:** Alessandra Pisciotta, Massimo Riccio, Gianluca Carnevale, Francesca Beretti, Lara Gibellini, Tullia Maraldi, Gian Maria Cavallini, Adriano Ferrari, Giacomo Bruzzesi, Anto De Pol

**Affiliations:** 1 Department of Surgical, Medical, Dental and Morphological Sciences with interest in Transplant, Oncology and Regenerative Medicine, University of Modena and Reggio Emilia, Modena, Italy; 2 Department of Biomedical, Metabolic and Neuroscience, University of Modena and Reggio Emilia, Children Rehabilitation Special Unit, IRCCS Arcispedale Santa Maria Nuova, Reggio Emilia, Italy; 3 Oro-Maxillo-Facial Department, AUSL Baggiovara, Modena, Italy; University of Udine, Italy

## Abstract

Human dental pulp is a promising alternative source of stem cells for cell-based tissue engineering in regenerative medicine, for the easily recruitment with low invasivity for the patient and for the self-renewal and differentiation potential of cells. So far, *in vitro* culture of mesenchymal stem cells is usually based on supplementing culture and differentiation media with foetal calf serum (FCS). FCS is known to contain a great quantity of growth factors, and thus to promote cell attachment on plastic surface as well as expansion and differentiation. Nevertheless, FCS as an animal origin supplement may represent a potential means for disease transmission besides leading to a xenogenic immune response. Therefore, a significant interest is focused on investigating alternative supplements, in order to obtain a sufficient cell number for clinical application, avoiding the inconvenients of FCS use. In our study we have demonstrated that human serum (HS) is a suitable alternative to FCS, indeed its addition to culture medium induces a high hDPSCs proliferation rate and improves the *in vitro* osteogenic differentiation. Furthermore, hDPSCs-collagen constructs, pre-differentiated with HS-medium *in vitro* for 10 days, when implanted in immunocompromised rats, are able to restore critical size parietal bone defects. Therefore these data indicate that HS is a valid substitute for FCS to culture and differentiate *in vitro* hDPSCs in order to obtain a successful bone regeneration *in vivo*.

## Introduction

Research in cell-based tissue engineering has been recently focused on identifying a cell source suitable for being applied in regenerative medicine. Dental pulp stem cells (DPSCs), discovered in human dental pulp even in young adults, are clonogenic cells capable of both self-renewal and multiple lineage differentiation [1]. DPSCs can be stimulated to differentiate, under specific conditions, towards various cell types, such as adipocytes, myoblasts, neurons, chondrocytes and osteoblasts [2]–[4]. Their wide range of differentiation allows them to represent a promising tool for reconstructive medicine, replacing lost or damaged tissues. This property may be related to the neural crest-cell origin of the dental pulp. It constitutes an adequate alternative source of stem cells for the easily recruitment with low invasivity for the patient and for the differentiation potential of its cells [5]–[6].

It has been shown that dental pulp stem cells proliferate and produce an extracellular matrix which subsequently becomes mineralized *in vitro* [5]. Recent studies have demonstrated that hDPSCs, after *in vitro* differentiation with an osteogenic medium, can differentiate into osteoblasts and produce mineralized extracellular matrix, both in 2D cultures and in 3D different biomaterials and, when implanted in immunocompromised animals produce bone and restore critical size bone defects [7]–[12].

*In vitro* culture of mesenchymal stem cells is based on supplementing cell culture and differentiation media with foetal calf serum (FCS). As it contains a great number of growth factors, FCS promotes cell attachment to plastic surfaces as well as cell proliferation and differentiation. Nevertheless, using FCS may trigger a xenogenic immune response and immunological reactions once cell transplantation in humans has been done [13]–[15]. This reason supports the attempt to evaluate MSCs culture and differentiation in media containing alternative supplements to FCS. Previous data report that human platelet lysate can replace FCS in terms of clinical-scale expansion [16]–[17] and *in vivo* bone-forming capacity of human stromal MSCs [18]. Felka *et al.* [19] demonstrated that human plasma represents a suitable replacement to FCS for expansion and differentiation of adipose derived stem cells. Human serum is also a suitable alternative, due to its availability and possibility for testing for human pathogens before use [17], [20].

In this study we have modified the expansion and differentiation media by replacing FCS with human serum (HS), to evaluate and compare the osteogenic phenotype developed by a sub-population of hDPSCs positive to STRO-1 antigen. In particular we analyzed the typical markers of the osteogenic differentiation, such as mineralized matrix production, alkaline phosphatase activity and osteopontin, osteocalcin, collagen type I expression. Finally, we have evaluated the hDPSCs ability of producing bone and restoring critical size bone defects when implanted *in vivo* in association with collagen scaffold.

## Materials and Methods

### Preparation of Human Serum

Human serum was obtained from a blood collection by healthy male volunteers who underwent written informed consent. The whole blood was drained into 8 ml tubes containing silica beads for clot activation (Greiner Bio-one, Kremsmünster, Austria), then was stored at room temperature for 3 hours and centrifuged at 1400×*g* for 15 minutes, to separate erythrocytes and coagulum contents. The collected serum was heat inactivated at 56°C for 30 minutes, then filtered with a 0.22 µm syringe-driven filters units (Millipore; Billerica, MA, USA). Aliquots of sterile human serum were stored at −20°C.

### Cell Culture

Human dental pulp was extracted from the enclosed third molar of teenage subjects undergoing a routine tooth extraction, after written informed consent of their parents. All procedures regarding collection of human samples were approved by the Provincial Ethics Committee of Santa Maria Nuova Hospital of Reggio Emilia. hDPSCs were isolated from dental pulp, as described in a previous study [8]. Briefly cells obtained from dental pulp were plated at clonal density (1.6 cells/cm^2^) and after 6 days of culture, nodules originated by single cells were isolated from the culture plate, re-plated and expanded. The STRO-1^+^ hDPSCs were obtained by magnetic cell sorting (MACS; Miltenyi Biotec) using an anti-STRO-1 antibody (Ab; Santa Cruz). Two different culture media were used during isolation steps and in the successive cultures: α-MEM supplemented with heat inactivated 10% Foetal Calf Serum (FCS-medium; Euroclone) or Human Serum (HS-medium), 2 mM L-glutamine, 100 U/ml penicillin, 100 µg/ml streptomycin, at 37°C and 5% CO_2_.

### Cell Counting

The proliferation rate was analyzed on the same hDPSCs population, seeded in 60 mm Petri dishes at the density of 4×10^3^ cells/cm^2^ and cultured for 1 week until reaching the confluence. Cell counting was performed in three culture conditions: serum free culture medium; culture medium supplemented with FCS; culture medium supplemented with HS. Each day cells were stained with CFDA (6-Carboxyfluoresceine diacetate, Sigma Aldrich; St. Louis, MO, USA) vital dye to detect viable cells and observed by using a Nikon TE2000 inverted microscope with a 10× objective. For each experimental point, green fluorescent cells contained in 10 fields of 1 mm^2^ were counted. The mean of cell number was calculated on three experimental samples for each condition and cell density was expressed as mean of cells/cm^2^± standard deviation (SD). The population doubling time (PDT) was calculated in the phase of exponential growth by the following formula:
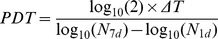



N_7*d*_ is the cell number at day 7 and N_1*d*_ is the cell number at day 1.

To determine the population doubling (PD) rate, hDPSCs were initially seeded at the density of 4×10^3^ cells/cm^2^ in culture medium supplemented with FCS or HS. Cells were passaged and counted once they reached a sub-confluence of 80%. At each passage cell were re-plated at the initial density and culture were performed until passage 5. Three samples for each condition were used. The following formula was applied:
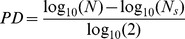



N is the harvested cell number and N_s_ is the initial plated cell number. Cumulative population doublings (CPD) index for each passage was obtained by adding the PD of each passage to the PD of the previous passages.

### Clonogenicity, Senescence and Cell Death

In order to evaluate colony forming ability, hDPSCs cultured in HS-medium and in FCS-medium were seeded at clonal density (1.6 cells/cm^2^) in 6-well plates and cultured for 10 days. Six samples for each culture condition were fixed in 4% paraformaldehyde in phosphate buffered saline (PBS) and stained with Toluidine blue. Counting was performed considering only colonies consisting of more than 10 cells.

In order to evaluate the presence of senescent cells in hDPSCs maintained in HS-medium and in FCS-medium, cells at 5^th^ passage were seeded in 12-well plates and cultured until reaching the confluence. Samples were then processed by a senescence β-Galactosidase staining kit (Cell Signaling), according to manufacturer’s instructions. Three samples for each culture condition were analyzed and percentage of senescent cells was calculated.

The presence of apoptotic cells in hDPSCs cultures was analyzed by detection of the cleaved form of poli(ADP-ribose)polymerase (PARP). Whole cell lysates of hDPSCs cultured in HS-medium and in FCS-medium at passages 1, 3 and 5 were processed for Western blot analysis and PARP was detected by an anti-PARP specific Ab (Santa Cruz).

#### FACS analysis

Isolated hDPSCs cultured for 5 passages in FCS-medium and HS-medium were assayed for the expression of Stro-1, c-Kit and CD34 surface antigens. Indirect staining was performed using mouse anti-STRO-1 IgM, rabbit anti-c-Kit IgG (Santa Cruz) and mouse anti-CD34 IgG (Millipore), followed by goat anti-mouse-IgG-Alexa488, goat anti-rabbit-IgG-Alexa488; and goat anti-mouse-IgM-Alexa488 (Invitrogen). Non-specific fluorescence was assessed by using normal mouse IgG or IgM followed by the secondary antibody as described above. Samples were analyzed using a 16-parameters CyFlow ML flow cytometer (Partec GmbH, Munster, Germany), equipped with a 488-nm blue solid-state, a 635-nm red diode laser, a UV mercury lamp HBO, a 532-nm green solid state laser, a 405-nm violet laser, and a CCD camera. Data were acquired in list mode by using FloMax (Partec) software, and then analyzed by FlowJo 9.4.11(Treestar Inc., Ash- land, OR) under MacOS 10. A minimum of 20,000 cells per sample were acquired.

### *In vitro* Multilineage Differentiation

hDPSCs cultured in HS-medium and FCS-medium were tested for their ability to differentiate towards Neurogenic, Adipogenic and Myogenic lineages. Three samples of each culture condition were used for each differentiation experiment.

Myogenic differentiation experiments were carried out according to Laino et al. [21]. For investigating the capability of hDPSCs to participate in myotubes formation, cells were seeded in direct co-culture with C_2_C_12_ mouse myoblast cell line. Human DPSCs and C_2_C_12_ cells were seeded in a 10∶1 ratio in DMEM High Glucose, supplemented with 10% FBS or HS, 2 mM L-glutamine, 100 U/ml penicillin, 100 µg/ml streptomycin, until confluence was reached. Upon confluence, growth medium was replaced with DMEM High Glucose supplemented with 1% FBS or HS and 10 nM insulin. Cells were maintained in co-culture for 2 weeks. Double immunofluorescence staining, by using an anti-human mitochondrial protein Ab (anti-hMit; Millipore, Billerica, MA, USA) and an anti-Myosin Ab (SIGMA), was performed in order to verify the formation of myotubes with the direct contribution of hDPSCs.

Neurogenic and Adipogenic differentiation were performed as described by Zang et al [22]. Neurogenic differentiation: cells were seeded on 6-well plates at 20,000 cells/cm^2^. After confluency was reached, cells were pre-induced with FCS-medium or HS-medium supplemented with 1 mM β-mercaptoethanol. After 24 h, the cells were washed and differentiated in serum free αMEM containing 10 mM β-mercaptoethanol, 2% dimethyl sulfoxide and 200 µM butylated hydroxyanisole until neuronal morphology was detectable. Neuronal differentiation was assayed for expression of β3-Tubulin by immunofluorescence experiments using specific Ab (Cell Signaling).

Adipogenic differentiation: cells were seeded on 24-well plates at 20,000 cells/cm^2^. Subconfluent cultures were incubated in the adipogenic medium (FCS-medium or HS-medium containing 0.5 mM isobutyl-methylxanthine, 1 µM dexamethasone, 10 µM insulin, 200 µM indomethacin, 50 mg/mL gentamicin) for 3 weeks. Medium was changed every 3 days. Afterwards, cells were stained with oil red O stain and counterstained with Harris haematoxylin.

### *In vitro* Osteogenic Differentiation

In order to obtain osteogenic differentiation on 2D surface, STRO-1^+^ hDPSCs were seeded at approximately 3×10^3^ cells/cm^2^ on culture dishes in two different osteogenic media (culture medium supplemented with 100 µM 2P-ascorbic acid, 100 nM dexamethasone, 10 mM β-glycerophosphate) containing 5% FCS (FCS-osteogenic medium) and 5% HS (HS-osteogenic medium), respectively. In particular hDPSCs cultured in HS-medium were differentiated in HS-osteogenic medium while cells cultured in FCS-medium were differentiated in FCS-osteogenic medium. The media were changed twice a week.

Collagen sponges (Condress, ABIOGEN PHARMA S.p.A.) were used as 3D scaffold in this study, for the subsequent *in vivo* implantation. Cells were seeded in collagen sponge samples (13 mm diameter; 1.5 mm height), into a 12 multiwell plate, in an adequate volume of culture medium to obtain a starting density of about 1×10^6^ cells per sponge. After 8 hours from cell seeding, 2 ml of the two osteogenic media were added to each sample. hDPSCs/collagen constructs were pre-differentiated for 10 days before *in vivo* implantation according to previous data [8], [10].

### Surgery and Implantation Procedure

The *in vivo* implantation has been realized in 14 weeks old Sprague-Dawley male rats and surgical procedure was performed as described by Riccio *et al.* [10].

In order to compare the bone forming ability of hDPSCs on collagen scaffolds in both pre-differentiation conditions, a critical size bone defect was generated by a full-thickness dissection of both the internal and external tables of compact bone constituting the parietal skeletal segment. The stem cell-scaffold complexes were then implanted into the critical size parietal defect up to 6 weeks.

One scaffold 5×8×1.5 mm size was implanted into each cranial defect and adapted to fill the entire defect area; each animal received two constructs: on the left parietal bone the construct pre-differentiated with FCS-osteogenic medium; on the right parietal bone the construct treated with HS-osteogenic medium. Control group received only collagen scaffold. Six weeks after the surgical implantation the rats were sacrificed and the calvarias were rapidly explanted and fixed in 4% paraformaldehyde in PBS for 3 h.

A total of 10 animals were used in this study (controls n = 4; treated n = 6). All animal procedures were performed according to the guidelines approved by the Committee of Use and Care of Laboratory Animals of the University of Modena e Reggio Emilia.

### Histology, Histochemistry and Morphometry

Some samples of *in vitro* differentiated hDPSCs were fixed in 4% paraformaldehyde in phosphate buffered saline (PBS) at pH 7.4 for 20 minutes and were processed for subsequent steps. For Alizarin red staining fixed cells were incubated for 5 minutes at room temperature in a solution containing 0,1% alizarin red and 1% ammonium hydroxide. Other samples were fixed in methanol-acetone 3∶7 for 10 minutes at −20°C, in order to perform the alkaline phosphatase assay. For this enzymatic assay fixed cells were processed by using Leukocyte Alkaline Phosphatase Kit (Sigma Aldrich; St. Louis, MO, USA), following the manufacturer’s instructions. Images were collected by a CCD colour camera equipped with a 90 mm macro photograph objective. Densitometry was performed on culture plates from three independent experiments by NIS software (Nikon). An equal area (ROI) was selected around the plate surface and the mean of gray levels (in a 0–256 scale) was calculated. Data were then normalized to values of background and expressed as mean ± SD.

Fixed samples of explanted calvarias were immersed in 0.5 M EDTA pH 8.3 until the complete decalcification and then rinsed in PBS, dehydrated with graded ethanol, diaphanized and embedded in paraffin. Transversal serial sections (10 µm thick) of the parietal bones containing the implants, were cut. Routine haematoxylin/eosin (H & E) staining was performed in order to analyze morphological details. Images of histological samples were obtained with a Nikon Labophot-2 optical microscope equipped with a DS-5Mc CCD color camera.

Morphometry on regenerating bone areas was performed by NIS software (Nikon). Implant area and new-formed bone area were measured in 5 transversal sections of implanted parietal bone for each animal. Percentage of new-formed bone was calculated respect to the total area of implant. Vasa, identified by morphological criteria and by labeling with anti-von Willebrand factor Ab, were counted in the area of not yet reabsorbed scaffold and in the area of new-formed bone for each animal and for each implant type. Data were normalized to areas of not yet reabsorbed scaffold and of new-formed bone respectively and were presented as mean ± SD of each experimental group (controls n = 4; treated n = 6).

### Confocal Miscroscopy

Fixed monolayer cells were permeabilized with 0,1% Triton X-100 in PBS for 5 minutes; samples were then blocked with 3% BSA in PBS for 30 minutes at room temperature and then incubated with the primary antibodies diluted 1∶50 [rabbit anti-osteocalcin (OCN), mouse anti-osteopontin (OPN), rabbit anti-osterix (Osx), rabbit anti-Runx2, mouse anti-hMit, rabbit-anti myosin, mouse anti β3-Tubulin] in PBS containing 3% BSA, for 1 hour at room temperature. After washing in PBS containing 3% BSA, the samples were incubated for 1 hour at room temperature with the secondary antibodies diluted 1∶200 in PBS containing 3% BSA (goat anti-rabbit FITC, sheep anti-mouse FITC, donkey anti-rabbit Cy3). After washing in PBS samples were stained with 1 µg/ml DAPI in PBS for 1 minute, and then mounted with anti-fading medium (0.21 M DABCO and 90% glycerol in 0.02 M Tris, pH 8.0).

Histological sections were processed as previously described by Riccio et al [10]. Primary antibodies (mouse anti-hMit, rabbit anti-OCN, Millipore; rabbit anti-von Willebrand factor, SIGMA) were diluted 1∶80 in PBS containing 3% BSA for 1 hour at room temperature. Secondary antibodies were diluted 1∶200 in PBS containing 3% BSA (goat anti-mouse alexa488, goat anti- rabbit alexa546, Life Technologies; Carlsbad, CA, USA). Negative controls were samples not incubated with the primary antibody. The multi-labelling immunofluorescence experiments were carried out avoiding cross-reactions between primary and secondary antibodies.

Immunohistochemistry with ant-hMit revealed by an anti-mouse HRP labelled secondary Ab (Pierce) was performed in order to confirm specificity of the labelling. HRP was revealed by a DAB based kit (SIGMA). Control without primary Ab was also performed.

Fluorescent samples were observed by a Nikon A1 confocal laser scanning microscope as described below. For detection, the samples were sequentially excited with the respective laser wavelength: 405 nm lines of a diode laser for DAPI; 488 nm lines of the argon laser for FITC or alexa488; 543 nm line of a HeNe laser for Cy3 or alexa546. The excitation and the detection of the samples were carried out in sequential mode to avoid overlapping of the two signals. Optical sections were obtained at increments of 0.3 µm in the z-axis and were digitized with a scanning mode format of 1024×1024 pixels and 4096 gray levels.

Spectral analysis was carried out to exclude overlapping between two signals or the influence of autofluorescence on the fluorochrome signals. Spectral imaging was carried out by simultaneous excitation of fluorochromes in the middle serial optical sections of the sample. Emission signals were acquired by a 32 line spectral detector and the full spectral emission was considered without any image elaboration. Moreover, ROIs were tracked on the images and the relative emission spectra were then analyzed. Spectra profiles were visualized in a wavelength range comprising the theoretical emission spectra of the fluorochromes present in the sample. In correspondence of the excitation laser wavelength the emission spectra show an unreal trend to zero because the correspondent line of the spectral detector was automatically turned off in order to avoid photomultiplier damages.

The confocal serial sections were processed with ImageJ software to obtain three-dimensional projections and image rendering was performed by Adobe Photoshop Software.

### Western Blot

Whole cell lysates were obtained at different times of differentiation by using an hypotonic buffer (30 mM Tris-Cl, pH 7.8, containing 1% Nonidet P40, 1 mM EDTA, 1 mM EGTA, 1 mM Na_3_VO_4_, and freshly added Sigma-Aldrich Protease Inhibitor Cocktail). Lysates were cleared by centrifugation and the total lysate was immediately boiled in SDS sample buffer. The protocols of the Western blot were performed as described by Sambrook et al [23]. Sixty µg of protein extract, quantified by a Bradford Protein Assay (Biorad), underwent SDS-polyacrylamide gel electrophoresis and were transferred to PVDF membranes. The following antibodies were used: rabbit anti-osteocalcin antibody diluted 1∶500; mouse anti-collagen type I (Millipore; Billerica, MA, USA), rabbit anti-PARP (Santa Cruz) diluted 1∶1000; peroxidase-labelled anti-rabbit and anti-mouse secondary antibodies diluted 1∶3000 (Pierce Antibodies, Thermo Scientific; Rockford, IL, USA). Antibody dilution was performed in TBS-T pH 7.6 containing 2% BSA and 3% free fatty milk. The membranes were visualized using ECL (enhanced chemioluminescence, Amersham, UK). Anti-actin antibody was used as control of protein loading. Densitometry of cleaved PARP bands was performed by NIS software (Nikon). An equal area was selected inside each band and the mean of gray levels (in a 0–256 scale) was calculated. Data were then normalized to values of background and of control actin band.

### Statistical Analysis

All experiments were performed in triplicate. Quantitative or semi-quantitative data were expressed as mean ± standard deviation (SD). Differences between two experimental points or between experiments were evaluated by paired Student's t-test. ANOVA test followed by Bonferroni post-test was used to analyze differences between three or more experimental points. In all analyses, values of *p*<0.01 were considered a significant statistical difference, while values of *p*<0.001 were considered a strong significant statistical difference.

## Results

### Cell Proliferation

**Figure 1:**
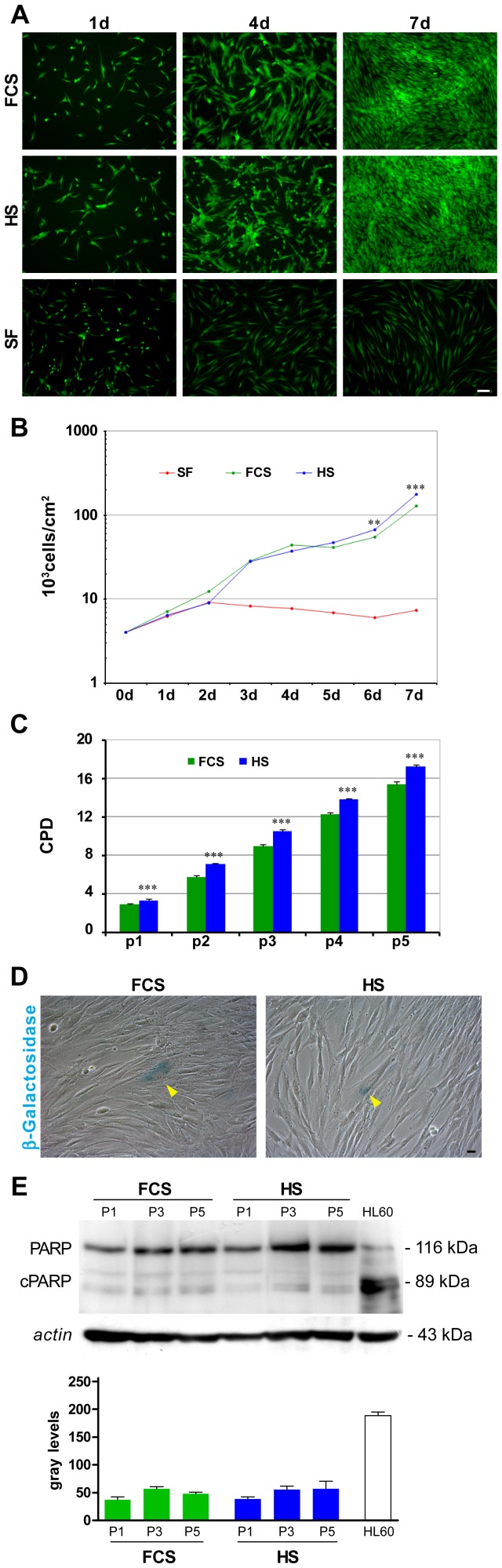
hDPSCs growing in FCS, HS and serum free (SF) media. A: CFDA vital staining of hDPSCs cultured for 1, 4 and 7 days. Green signal indicates viable cells. Bar: 100 µm. B: Proliferation rate of hDPSCs cultured for a week; Values are mean reported in a Log scale, n = 3; * indicates values of paired *t*-test HS vs. FCS (**p<0.01, ***p<0.001). C: Cumulative population doubling (CPD) of hDPSCs cultured for a total of 5 passages. At each passage cells cultured in HS-medium show a CPD significantly higher than FCS-medium cultured cells (n = 3; ***p<0.001). D shows β-galactosidase activity staining in confluent culture of hDPSCs grown for 5 passages in FCS-medium or in HS-medium as indicated. Arrowheads indicate cells positive to β-galactosidase activity staining. Bar 10 µm. E: western blot analysis of PARP in hDPSCs cultured in FCS-medium and in HS-medium at passages 1, 3 and 5. HL60, treated with etoposide, were loaded as positive control of the presence of cleaved PARP (cPARP). Actin bands were presented as control of the protein loading. Densitometry of cPARP bands was shown on the bottom of western blot images.

To evaluate the influence of HS or FCS on the growth of hDPSCs during routine cell culture, proliferation rate was evaluated by using 6-CFDA staining to detect viable cells in adherent culture at different times until confluence was reached. hDPSCs were then grown in culture media without serum or containing respectively 10% FCS and 10% HS, and cell counting was performed for up to 7 days (Fig. 1A). Human DPSCs cultured in serum free medium show a not significant proliferation rate indicating a stationary trend. Cells cultured with HS show, at the beginning, a slightly lower proliferation rate compared to hDPSCs growing in FCS-medium. From day 4 onward the proliferation rate of cells in HS-medium slightly exceeds, in significant manner, the one of hDPSCs growing in FCS-medium (Fig. 1B). Furthermore the population doubling time of HS cultures is lower than FCS cultures (HS: 35.0±2.31 hours; FCS: 40.3±1.50 hours; *p*<0.01). Cumulative population doubling analysis shows that HS culture proliferates more speedily, overtaking FCS culture of about 13% at each passage (Fig. 1C).

hDPSCs cultured in HS-medium or in FCS-medium for 5 passages were seeded at clonal density and cultured in 6-well culture plates for 10 days in order to verify differences in clonogenic ability. No significant differences were observed in the number of colonies formed by hDPSCs growing in the two culture conditions (HS 17.3±4.8 colonies; FCS 18.7±4.0 colonies).

### Senescence and Apoptosis

Cell senescence was evaluated by detection of β-galactosidase activity in confluent culture of hDPSCs grown for 5 passages in HS-medium or in FCS-medium. Very low levels of β-galactosidase activity were detected in both culture conditions (Fig. 1D arrowheads). In particular no significant differences were observed in the percentage of senescent cells detected by microscopic observation in the two culture conditions (FCS 0.044±0.007%; HS 0.039±0.004%; n = 3).

Western blot analysis of PARP carried out in hDPSCs at passages 1, 3 and 5, shows an equivalent amount of uncleaved PARP (116 kDa) in both culture conditions. On the other hand positive control, (etoposide treated HL60 cell line) shows a clear band in correspondence of cleaved PARP form (89 kDa; Fig. 1E) that is not appreciable in hDPSCs. Densitometry performed on the cleaved PARP bands did not show significant differences between HS and FCS cultured DPSCs.

These data indicate that HS-medium does not induce premature senescence and does not affect the survival of hDPSCs.

### Surface Antigens Expression and *in vitro* Multilineage Differentiation

In order to verify if HS influences stemness of hDPSCs during *in vitro* culture, surface antigens expression and multilineage differentiation ability were evaluated in hDPSCs cultured in HS-medium and in FCS-medium.

**Figure 2:**
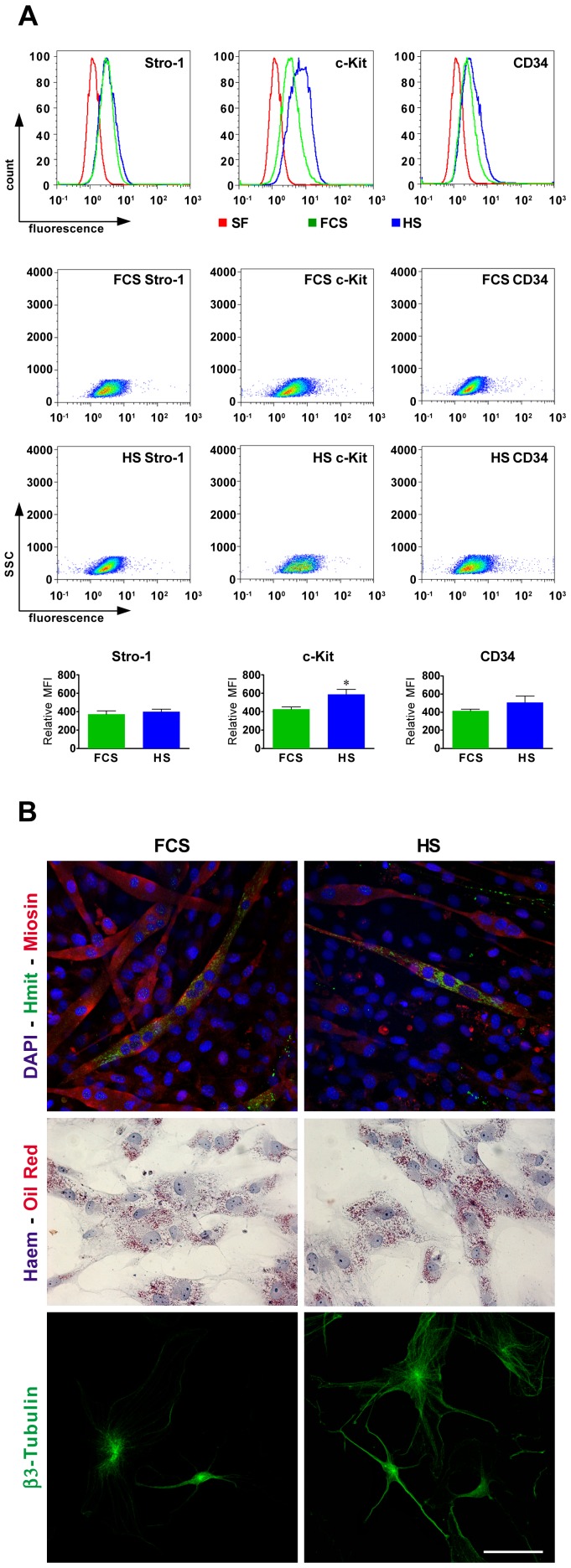
hDPSCs surface antigens expression and *in vitro* multilineage differentiation ability. A: Cytofluorimetric analysis of Stro-1, c-Kit and CD34 expression in hDPSCs cultured in FCS-medium and in HS-medium. Dot plots reporting SSC vs fluorescence are shown. In the histograms, the net fluorescence value was calculated by linearizing the fluorescence value from the logarithmic scale and subtracting the linearized value of the unstained sample to the linearized value of the stained one. Data represent the mean±SD of three different experiments. * indicates values of paired *t*-test HS vs. FCS (*p<0.05). B: In the first line are shown double immunofluorescence images of hDPSCs/C_2_C_12_ co-culture stained by anti-hMit (green) and anti-myosin (red) Abs. DAPI staining is shown in blue. The second line shows oil red staining of hDPSCs differentiated for two weeks towards adipogenic lineage with HS or FCS supplemented medium. Cells were counterstained with Harris haematoxylin. Images, in third line represent anti-β3-Tubulin immunofluorescence labelling on hDPSCs differentiated in culture neurogenic media supplemented with FCS or HS. Bar 50 µm.

Stro-1, c-Kit, and CD34 are surface antigens typical of hDPSCs [21]. FACS analysis performed in hDPSCs in both culture conditions after 5 passages, demonstrates that hDPSCs maintain the expression of the above mentioned antigens. Moreover, the fluorescence intensity of c-Kit labelling appears higher in HS-cultures, indicating that the whole population expresses this antigen at a dim level. Stro-1 and CD34 antigens appear equally expressed (Fig. 2A).

hDPSCs cultured in both media were differentiated towards myogenic, adipogenic, neurogenic and osteogenic lineages as described in materials and methods.

The ability of hDPSCs to differentiate towards myogenic lineage was verified by co-culture with C_2_C_12_ mouse myoblasts. After 14 days of co-culture myotubes formation was observed both in HS co-cultures and in FCS co-cultures. Myotubes appear multi-nucleated indicating that cell fusion occurs. Labelling by anti-human mitochondria antibody (anti-hMit) demonstrates that hDPSCs are involved in myotubes generation. Double staining with anti-hMit and anti-myosin antibodies indicates that in both culture conditions mature myotubes were formed with the contribution of hDPSCs. Myotubes not labelled by anti-hMit antibody and therefore formed only by C_2_C_12_ cells were also present (Fig. 2B). The percentage of myotubes formed with the contribution of hDPSCs respect to the total myotubes, was similar in both culture conditions (FCS 42,7±6,4%; HS 46,3±7,2%; n = 3).

hDPSCs cultured in adipogenic medium supplemented with HS start to form intracellular lipidic drops after three days of differentiating culture while cells differentiating in FCS adipogenic medium show the first intracellular lipidic drops at the 7^th^ day. After two weeks of differentiation, hDPSCs in both conditions show a similar morphology characterized by numerous lipidic drops accumulated in the cytoplasm and clearly stained by oil red (Fig. 2B). The percentage of oil Red positive cells did not show significant differences between the two culture conditions (FCS 73,7±8,1%; HS 75,7±9,0%; n = 3).

Neurogenic differentiation starts with an initial cell detachment from culture plates probably induced by β-mercaptoethanol. Remaining cells progressively assume neuronal morphology with multiple cellular processes and a defined cell body. Immunostaining with anti-β3-Tubulin after 20 days of culture shows the presence of neurotubules in hDPSCs pre-differentiated in FCS-medium as well as in HS-medium (Fig. 2B). No particular differences, in the percentage of β3-Tubulin positive cells, were observed between the two experimental groups (FCS 64,3±7,2%; HS 62,0±7,6%; n = 3).

**Figure 3:**
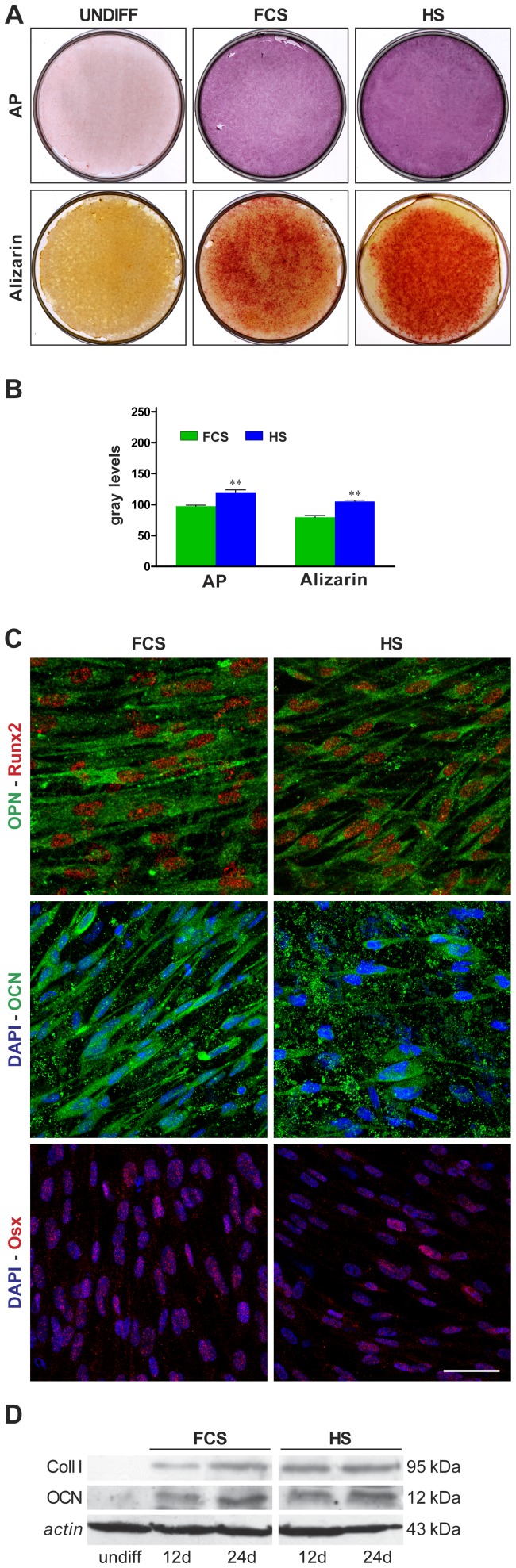
*In vitro* osteogenic differentiation of hDPSCs after 24 days of culture in osteogenic medium, supplemented with FCS or HS. A: Alkaline phosphatase assay (AP) and Alizarin Red staining; B: densitometric analysis of AP and Alizarin Red staining. Values are mean ± SD of gray levels (0–255 scale). HS n = 3; FCS n = 3; ** indicate values of paired t-test HS vs. FCS (*p<*0.01). C: Confocal analysis of osteogenic differentiation of hDPSCs. Double immunofluorescence confocal images showing signals from anti-OPN (green) and anti-Runx2 (red); DAPI (blue) and anti-OCN (green); DAPI (blue) and anti-Osx (red). Bar: 50 µm. D: Western blot (WB) analysis of Coll-I and OCN expression in whole cell lysates of differentiated hDPSCs. Whole cell lysates were collected from three plates of human dental pulp stem cells for each differentiation protocol. Actin bands demonstrate that an equal amount of protein was loaded in each line. Undiff samples show hDPSCs cultured in FCS-medium alone. The same results were obtained in HS-medium culture (data not shown).

Osteogenic differentiating hDPSCs start to form nodular aggregates at day 8. At day 24, mineral deposits appear in the extracellular space and in nodular aggregates (data not shown). In cells, both the differentiation conditions induce an intense positivity to Alkaline phosphatase assay, which is extended to the whole culture plate. The *in vitro* deposition of mineralized extracellular matrix was analyzed by Alizarin red staining: differences were observed in Alizarin red staining, in favour of hDPSCs treated with HS (Fig. 3A), which show a greater deposition of mineralized extracellular matrix than hDPSCs differentiated with FCS, as confirmed by densitometric analysis. A higher alkaline phosphatase activity was detected in HS-treated cells (Fig. 3B). Controls of undifferentiated cells are negative for both the stainings (Fig. 3A).

The confocal analysis at day 24 shows the expression of osteocalcin, together with other specific markers of osteogenic commitment, such as osteopontin (OPN), osterix (Osx) and Runx2. Double immunofluorescence labelling was carried out to analyze simultaneously the localization of OPN and Runx2 in hDPSCs differentiated with FCS-medium or HS-medium (Fig. 3C). OPN appears localized in the cytoplasm perinuclear region, that normally contains the rough endoplasmic reticulum, while Runx2 is localized in the cell nucleus as expected. OCN shows a typical cytoplasmic localization, strongly expressed in both the differentiation conditions. Signal from OCN was also detected in extracelluar matrix where it appears as spot localized in mineralization areas. Similarly to Runx2, Osx shows the typical nucleoplasmic localization, as demonstrated by the overlapping with DAPI signal (Fig. 3C). No differences were observed between the two differentiation conditions.

To confirm the differentiation of hDPSCs towards osteoblast-like cells, in both osteogenic media, the presence of type I collagen (Coll-I) and of the specific marker osteocalcin (OCN), was analyzed by Western blot in whole cell lysates of differentiating hDPSCs: Coll-I and OCN were detected in both the differentiating conditions, and show an increase of expression during the differentiation (Fig. 3D).

### *In vivo* Osteogenic Differentiation

In order to evaluate if the use of different sera (FCS and HS) during pre-differentiation of hDPSCs seeded in collagen scaffold, influences differently their ability to reconstruct critical size parietal bone defects, *in vivo* implants were performed as described in materials and methods section. After six weeks parietal bones containing the implants were histologically processed and observed by white field microscopy.

**Figure 4:**
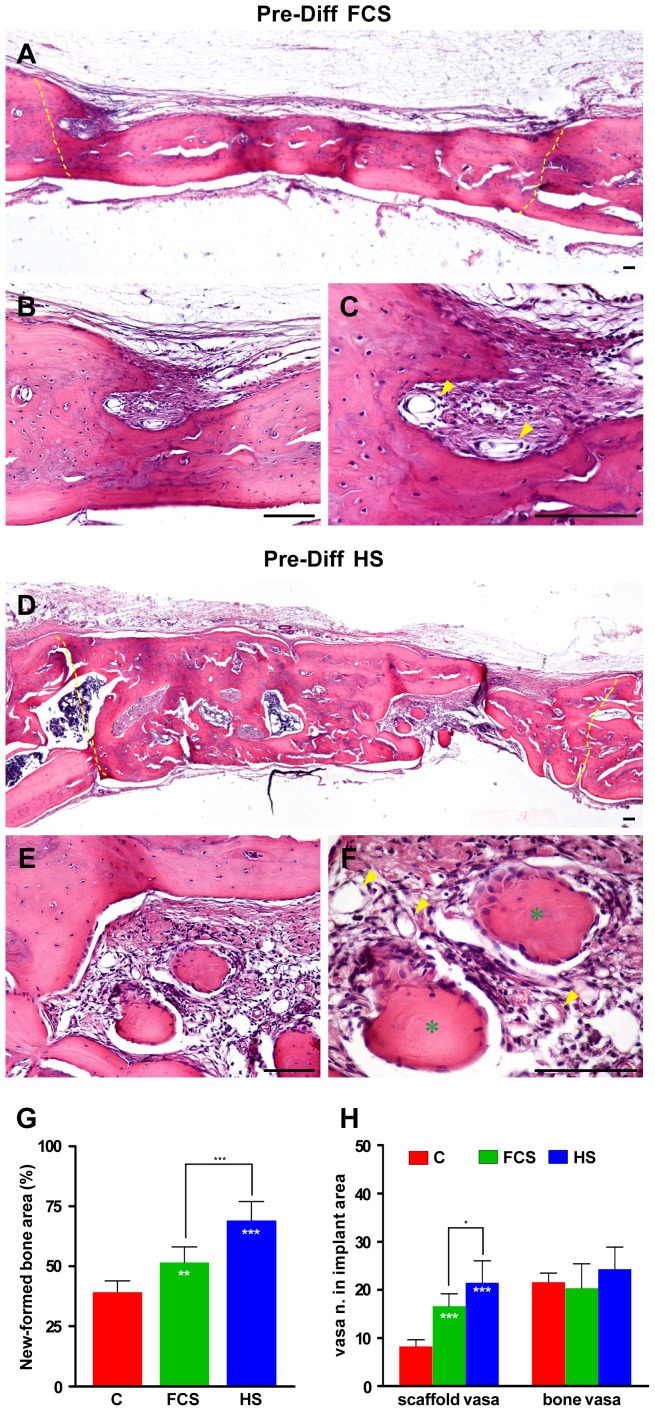
Comparative histological analysis (haematoxylin/eosin staining) of the critical size cranial defect reconstruction by hDPSCs/collagen constructs 40 days post-surgery. Images show transversal sections carried out through the central area of the implants. A–C: cranial defect filled with hDPSCs/collagen pre-differentiated with FCS containing medium; D–F: cranial defect closed with hDPSCs/collagen pre-differentiated with HS containing medium; (dotted line delimitates the areas of bone resection; arrowheads indicate vasa; * indicate areas of active bone deposition). Bar: 100 µm. G: morphometric analysis of new-formed bone areas in controls (C), FCS and HS implants. Values are mean ± SD of the percentage of regenerated bone respect to the whole resected bone area. HS and FCS n = 6; C n = 4; white * inside the column indicate values of ANOVA test of HS and FCS vs. C (**p<0.01, ***p<0.001); black * indicate values of ANOVA test of HS vs. FCS (***p<0.001). H: number of vasa in the scaffold not yet reabsorbed and in new-formed bone areas. Data, normalized to areas of the scaffold not yet reabsorbed and of new-formed bone areas respectively, were presented as mean ± SD (vasa number respect to the total implant area) of each experimental group (controls n = 4; treated n = 6). White * inside the column indicate values of ANOVA test of HS and FCS vs. C (***p<0.001); black * indicate values of ANOVA test of HS vs. FCS (*p<0.05).

Figure 4 shows representative photomicrographs of engineered bone grafts *in vivo*.

The hDPSCs/collagen constructs realized in both pre-differentiation conditions exhibit an appreciable contribution to regeneration of the resected bone area (Fig. 4A, 4D). Magnification images show the presence of vessels indicating a successful vascularization of the implants (Fig. 4: B–C–E–F, arrowheads) and areas of active bone deposition and rearrangement, particularly detectable in samples pre-differentiated with osteogenic HS-medium, where osteoblast layer surrounds islets of new-formed bone (Fig. 4E, 4F, star). Morphometric analysis was carried out in order to evaluate the amount of new-formed bone areas in animals implanted with collagen scaffold colonized with FCS or HS pre-differentiated hDPSCs and in the control group implanted only with the collagen scaffold. The analysis revealed that animals implanted with scaffold colonized with hDPSCs pre-differentiated in FCS and in HS differentiating medium show a bone regeneration significantly higher when compared to the control group (FCS vs C *p*<0.01; HS vs C *p<*0.001; Fig. 4G). Moreover, by comparing HS implants with FCS implants emerges that the new-formed bone area is greater in HS implants respect to the FCS implants (HS vs FCS *p<*0.001; Fig. 4G). The number of vasa in the scaffold not yet reabsorbed appears higher in HS pre-differentiated cells suggesting a better angiogenic potential. On the other hand the number of vasa in new-formed bone does not show significant differences among the three experimental groups indicating that the new-formed bone structure was not affected by the different pre-differentiation conditions (Fig. 4H).

**Figure 5:**
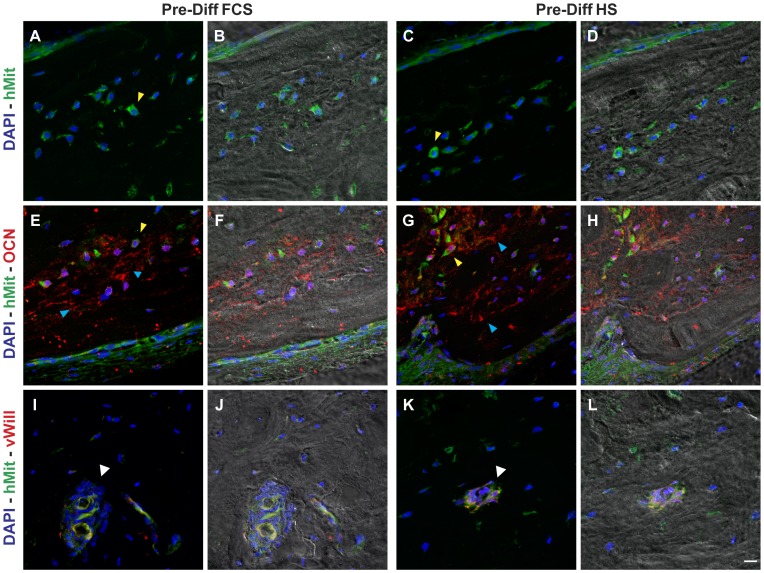
Confocal images of implants pre-differentiated in both the conditions. A–D: Double fluorescence signals from DAPI (blue) and anti-hMit Ab (green) superimposed to pseudo-phase contrast images (B, D). Arrowheads indicate cells entrapped in calcified bone matrix clearly stained by anti-human mitochondria antibody. E–H: triple fluorescence signals from DAPI (blue) and anti-hMit (green) and anti-OCN (red) Abs superimposed to pseudo-phase contrast images (F, H). Yellow arrowheads indicate osteocytes labelled by the two Abs; cyan arrowheads indicate OCN deposits in extracelluar bone matrix. I–L: triple fluorescence signals from DAPI (blue) and anti-hMit (green) and anti-von Willebrand factor (red) Abs superimposed to pseudo-phase contrast images (J, L). Arrowheads indicate vasa double stained by the two Abs. Bar: 10 µm.

To verify if cells inside the new-formed bone were still from human origin and not from the host, immunofluorescence labelling with anti-hMit antibody was performed. This antibody recognizes only mitochondrial protein from human origin: images of stem cell-scaffold implants demonstrate that most of the cells are clearly stained by anti-human mitochondria antibody (Fig. 5 A–D) indicating survival of the donor cells *in vivo,* in both the pre-differentiation conditions. Specificity of anti-hMit immunofluorescence labelling was demonstrated in supporting information (Fig. S1 A–B, E–F) as well as immunoperoxidase reaction confirms the presence of human cells inside the new-formed bone (Fig. S1 C–D).

Interestingly, human DPSCs are included within bone lacunae indicating the direct contribution to the osteocytes population of new-formed bone. Double immunofluorescence analysis shows that human cells express OCN that was detectable also in new-formed bone matrix (Fig. 5 E–H). Moreover cells localized in the inner layer of vascular canals show a clear staining by anti-human mitochondria antibody, suggesting a direct contribution of hDPSCs to the angiogenetic process that originates endothelial cells of new-formed vasa. Double labelling by anti-hMit and anti-von Willebrand factor indicates that hDPSCs contribute to the constitution of the endothelium of vasa localized in the new-formed bone (Fig. 5 I–L).

Spectral analysis demonstrates the specificity of the double immunofluorescence labelling (Fig. S1 G–H).

## Discussion

In this study we have investigated a culture condition that may allow human DPSCs for cell-based therapy to be used in clinical application with minimal safety concerns. This goal assumes it is desirable and necessary to obtain a sufficient number of cells, as the result of expansion and differentiation conditions realized by using the most suitable reagents to promote cell proliferation and differentiation. FCS has been widely used for this purpose over the past several decades. Anyway, some potential hazards related to using FCS in clinical settings have been raised in terms of potential disease transmission and xenogenic immunologic response [24]. Indeed previous works indicate that FCS proteins can be internalized in stem cells and involve the hazard of pathogen transmission: bovine proteins may also be recognized as antigenic substrates, leading to a xenogenic immune response [14]–[15]. For these reasons, in addition to good manufacturing practices (GMP), clinical protocols for cell therapy encourage the use of FCS substitute [25].

Even serum free media have been tested, but so far literature has demonstrated these media do not constitute an adequate support for proliferation of mesenchymal stem cells, without addition of exogenous growth factors [26], [27]. Supplementation of media with small amount of serum and specific factor as EGF and PDGF could represent a valid perspective [28].

Various human plasma derivatives have been purposed as alternatives to FCS to supply nutrients, adhesion and growth factors. These alternative supplements include autologous or allogenic human serum, human plasma, human platelet lysates and their released factors [16], [20], [29]. In the last years platelet-rich plasma was largely used to growth and differentiate MSC but its role in MSC osteogenic differentiation is debated [30]–[32]. The use of human serum, preferably autologous, appears to provide benefits for different aspects in culture and differentiation of human synovial MSCs and human bone marrow MSCs [33]–[35]. Evidence in favour of using autologous serum also exist for the expansion of human bone marrow MSCs, indeed it results as effective as supplementing culture medium with FCS, and cells appear transcriptionally more stable over many cell doublings [14]. Nevertheless, autologous serum may also be a scarcely available or qualitatively affected source, particularly in the case it must be taken from some classes of patients, such as children, elderly, anaemic and with ongoing inflammatory processes individuals [33]. In these cases, allogenic human serum, potentially from blood type AB healthy donors may represent a preferable alternative to autologous serum.

To date, human platelet lysate and platelet-rich plasma have been shown to be suitable culture supplements to replace FCS, for the expansion and *in vitro* osteogenic differentiation of hDPSCs [36], [37]. To the best of our knowledge, in literature there is not yet evidence that human serum may constitute a suitable replacement to FCS for culture and differentiation of human DPSCs.

Our data demonstrate that HS is an appropriate supplement for the *in vitro* expansion of human DPSCs, as its addition to culture medium promotes a good proliferation rate comparable to the one measured in culture conditions using FCS. Furthermore, HS increases the hDPSCs doubling during culture indicating this as an the optimal condition to obtain sufficient cell number soon and consequently to reduce the waiting for a therapeutical treatment. Otherwise, cells cultured in serum free medium were growth arrested. Moreover HS does not induce cell senescence or apoptosis and does not affect the surface antigens expression and the multi-lineage differentiation ability of hDPSCs.

The expression of bone related proteins as well as the mineralization of the extracellular matrix analyzed in *in vitro* osteogenic differentiation experiments indicate that the use of HS is adequate to hDPSCs osteogenic differentiation protocols. Moreover HS, respect to FCS, seems to induce a better mineralization of extracellular matrix synthesized by differentiating hDPSCs.

When transplanted *in vivo*, in immunocompromised animals, by means of stem cell-scaffold constructs, hDPSCs pre-differentiated in both the osteogenic media show a significant contribution to the regeneration of critical size bone defect. Immunofluorescence staining with anti-human mitochondria antibody demonstrates that hDPSCs widely contribute to the osteocyte population of the new-formed bone and seem to participate to the angiogenetic process that gives rise to endothelial cells of the new-formed bone.

Thus, in our study we have demonstrated that human serum represents an eligible alternative to FCS, for successfully expanding and differentiating human DPSCs towards osteogenic lineage, finding, furthermore, a helpful application for *in vivo* transplantation. The results we have obtained *in vivo* allow to propose human serum as additive for expansion and differentiation of hDPSCs to be applied in human cell therapy with GMP-compatible protocols. Indeed, it matches with the criteria approved by the Committee for medicinal products for human use.

## Supporting Information

Figure S1 A–B: DAPI/anti-hMit immunofluorescence staining on control implant (only collagen sponge). C: immunohistochemical staining with anti-hMit Ab on section from implants pre-differentiated with HS. Black arrowheads indicate cells positive to anti-hMit Ab. Red arrowhead indicates vasa with endothelium stained by anti-hMit Ab. D: control; immunoperoxidase was carried out without primary Ab. E: Spectral image of immunofluorescence staining on control implant not containing human cells. Image was acquired on the same field of fig. A–B. ROI indicates areas of spectral emission analysis of DAPI and Alexa488 fluorochromes, reported with same color in the graphs on the right side. E1 shows theoretical emission spectra of DAPI superimposed to DAPI emission from sample. E2 represents emission of Alexa488 from sample superimposed on their theoretical spectrum.F: Spectral image of immunofluorescence staining with anti-hMit Ab counterstained with DAPI. ROI indicates areas of spectral emission analysis of DAPI and Alexa488 fluorochromes, reported with same color in the graphs on the right side. In the graphs are reported theoretical emission spectra of DAPI and Alexa488 superimposed to the emission of: DAPI from sample (E1), Alexa488 from sample (E2). Signals from DAPI and Alexa488 match with respective theoretical spectra. G: Spectral image of double immunofluorescence staining with anti-hMit and anti-OCN Abs. ROI indicated areas of spectral emission analysis reported with same color in the graphs on the right side. In the graphs were reported theoretical emission spectra of Alexa488 and Alexa546 superimposed to the emission of Alexa488, Alexa546. G1 represents the emission of double signal from a cell double labelled by anti-hMit and anti-OCN Abs revealed by Alexa488 and Alexa546 respectively. G2 describes the spectral emission of the area containing OCN extracellular deposits that emit a signal matching with Alexa546 theoretical spectrum. H: spectral image of signals from anti-hMit (green) and anti-von Willebrand factor (red). ROI in the image represents areas analyzed in the graphs on the right side. Corresponding spectra are shown in the same color of ROI. H1 represents emission spectra of area labelled with anti-hMit and anti-von Willebrand factor Abs revealed by Alexa488 and Alexa546 respectively. Emission spectrum from sample presents two peaks overlapping the theoretical spectra of both Alexa488 and Alexa546. Arrows on the top of all graphs indicate the wavelengths of laser lines used to excite the respective fluorochromes. In correspondence of the excitation laser wavelength the emission spectra show an unreal trend to zero since the correspondent line of the spectral detector was automatically turned off in order to avoid photomultiplier damages. Images show implants of hDPSCs pre-differentiated with HS-medium. The same results were obtained with FCS-medium (data not shown). th: theoretical; Alx: Alexa.
(TIF)
