# Supplementary figures and images for: Human Serum Promotes Osteogenic Differentiation of Human Dental Pulp Stem Cells In Vitro and In Vivo
Source: PLoS One. 2012 Nov 29;7(11):e50542. doi: 10.1371/journal.pone.0050542 (PMC3510089; doi:10.1371/journal.pone.0050542)

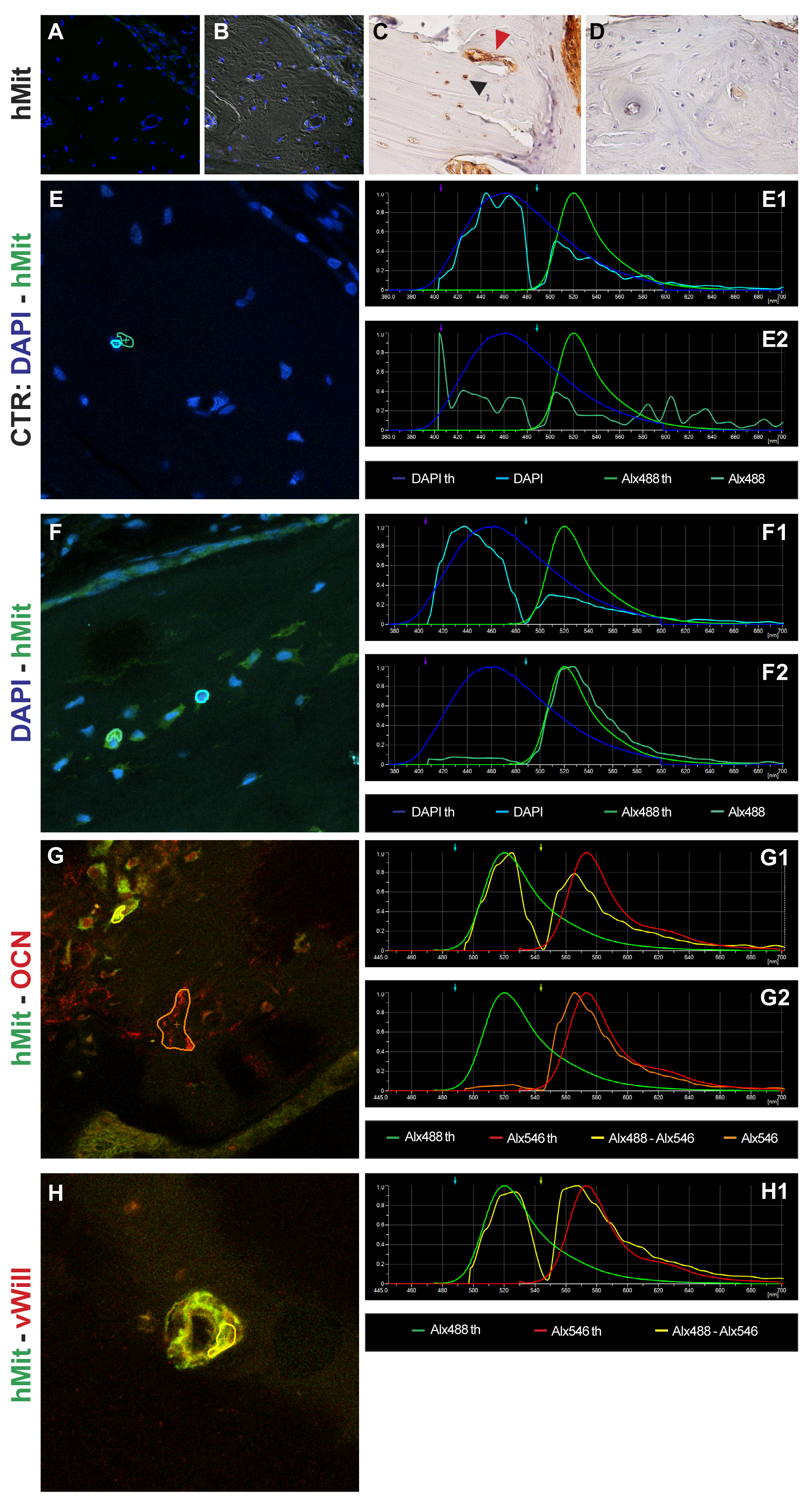

Supplement: Figure S1 — A–B: DAPI/anti-hMit immunofluorescence staining on control implant (only collagen sponge). C: immunohistochemical staining with anti-hMit Ab on section from implants pre-differentiated with HS. Black arrowheads indicate cells positive to anti-hMit Ab. Red arrowhead indicates vasa with endothelium stained by anti-hMit Ab. D: control; immunoperoxidase was carried out without primary Ab. E: Spectral image of immunofluorescence staining on control implant not containing human cells. Image was acquired on the same field of fig. A–B. ROI indicates areas of spectral emission analysis of DAPI and Alexa488 fluorochromes, reported with same color in the graphs on the right side. E1 shows theoretical emission spectra of DAPI superimposed to DAPI emission from sample. E2 represents emission of Alexa488 from sample superimposed on their theoretical spectrum.F: Spectral image of immunofluorescence staining with anti-hMit Ab counterstained with DAPI. ROI indicates areas of spectral emission analysis of DAPI and Alexa488 fluorochromes, reported with same color in the graphs on the right side. In the graphs are reported theoretical emission spectra of DAPI and Alexa488 superimposed to the emission of: DAPI from sample (E1), Alexa488 from sample (E2). Signals from DAPI and Alexa488 match with respective theoretical spectra. G: Spectral image of double immunofluorescence staining with anti-hMit and anti-OCN Abs. ROI indicated areas of spectral emission analysis reported with same color in the graphs on the right side. In the graphs were reported theoretical emission spectra of Alexa488 and Alexa546 superimposed to the emission of Alexa488, Alexa546. G1 represents the emission of double signal from a cell double labelled by anti-hMit and anti-OCN Abs revealed by Alexa488 and Alexa546 respectively. G2 describes the spectral emission of the area containing OCN extracellular deposits that emit a signal matching with Alexa546 theoretical spectrum. H: spectral image of signals from anti-hMi [file pone.0050542.s001.tif]
